# Vasodilator-Stimulated Phosphoprotein (VASP)-dependent and -independent pathways regulate thrombin-induced activation of Rap1b in platelets

**DOI:** 10.1186/s12964-016-0144-z

**Published:** 2016-09-13

**Authors:** Peter M. Benz, Hebatullah Laban, Joana Zink, Lea Günther, Ulrich Walter, Stepan Gambaryan, Karim Dib

**Affiliations:** 1 Institute for Vascular Signalling, Centre for Molecular Medicine, Johann Wolfgang Goethe University and DZHK (German Centre for Cardiovascular Research) partner site Rhine-Main, 60590 Frankfurt, Germany; 2 Centre for Thrombosis and Hemostasis (CTH), University Medical Center Mainz, Mainz, Germany; 3 Department of Cytology and Histology, St. Petersburg State University, St. Petersburg, Russia; 4 Sechenov Institute of Evolutionary Physiology and Biochemistry, Russian Academy of Sciences, St. Petersburg, Russia; 5 Centre for Experimental Medicine, Medical Biology Center (MBC) building, Queen’s University of Belfast, Third floor, 97 Lisburn Road, BT9 7BL Belfast, Northern Ireland UK

**Keywords:** Platelets, VASP, Rap1b, Crkl, cAMP, cGMP

## Abstract

**Background:**

Vasodilator-Stimulated Phosphoprotein (VASP) is involved in the inhibition of agonist-induced platelet aggregation by cyclic nucleotides and the adhesion of platelets to the vascular wall. α_IIb_β_3_ is the main integrin responsible for platelet activation and Rap1b plays a key role in integrin signalling. We investigated whether VASP is involved in the regulation of Rap1b in platelets since VASP-null platelets exhibit augmented adhesion to endothelial cells in vivo.

**Methods:**

Washed platelets from wild type and VASP-deficient mice were stimulated with thrombin, the purinergic receptors agonist ADP, or the thromboxane A2 receptor agonist U46619 and Rap1b activation was measured using the GST-RalGDS-RBD binding assay. Interaction of VASP and Crkl was investigated by co-immunoprecipitation, confocal microscopy, and pull-down assays using Crkl domains expressed as GST-fusion proteins.

**Results:**

Surprisingly, we found that activation of Rap1b in response to thrombin, ADP, or U46619 was significantly reduced in platelets from VASP-null mice compared to platelets from wild type mice. However, inhibition of thrombin-induced activation of Rap1b by nitric oxide (NO) was similar in platelets from wild type and VASP-null mice indicating that the NO/cGMP/PKG pathway controls inhibition of Rap1b independently from VASP. To understand how VASP regulated Rap1b, we investigated association between VASP and the Crk-like protein (Crkl), an adapter protein which activates the Rap1b guanine nucleotide exchange factor C3G. We demonstrated the formation of a Crkl/VASP complex by showing that: 1) Crkl co-immunoprecipitated VASP from platelet lysates; 2) Crkl and VASP dynamically co-localized at actin-rich protrusions reminiscent of focal adhesions, filopodia, and lamellipodia upon platelet spreading on fibronectin; 3) recombinant VASP bound directly to the N-terminal SH3 domain of Crkl; 4) Protein Kinase A (PKA) -mediated VASP phosphorylation on Ser157 abrogated the binding of Crkl.

**Conclusions:**

We identified Crkl as a novel protein interacting with VASP in platelets. We propose that the C3G/Crkl/VASP complex plays a role in the regulation of Rap1b and this explains, at least in part, the reduced agonist-induced activation of Rap1b in VASP-null platelets. In addition, the fact that PKA-dependent VASP phosphorylation abrogated its interaction with Crkl may provide, at least in part, a rationale for the PKA-dependent inhibition of Rap1b and platelet aggregation.

## Plain English summary

Platelets, or thrombocytes, are the second most common kind of blood cells in the human body. Along with the coagulation factors, the main function of platelets is to stop bleeding by clumping and clotting blood vessel injuries (hemostasis). Hemostasis can be divided into three parts: platelet binding to the injured vessel (adhesion), platelet shape change and secretion of messengers (activation), and platelet bridging (aggregation). An important step in platelet function is the activation of the platelet receptor integrin α_II__b_β_3_, which is regulated by the small GTPase Rap1b. In the present study, we investigated whether Vasodilator-Stimulated Phosphoprotein (VASP) is involved in the regulation of Rap1b in platelets. To address this question, we activated platelets from wild-type and VASP-deficient mice and measured Rap1b activity. We found that activation of Rap1b was reduced in platelets from VASP-deficient mice. We identified a novel VASP binding partner in platelets, the adapter protein Crkl, which is important for Rap1b activation. Furthermore, we found that the interaction of Crkl and VASP is abrogated by PKA, which plays an important role in the inhibition of platelet function. Together, we propose that the control of the interaction of VASP and Crkl by PKA is important for Rap1b activation in platelets.

## Background

The central event in platelet adhesion at sites of vascular injury is the agonist-induced inside-out activation of α_IIb_β_3_ integrin [1], a process in which the small GTPase Rap1b plays a key role [2]. Indeed, Rap1b is activated in response to agonists [3] and platelets from Rap1b knockout mice exhibit defects in αIIbβ3 integrin-dependent arterial thrombus formation [4]. Rap1b cycles between an active GTP-bound and an inactive GDP-bound conformation. The GDP-GTP switch is brought about by the guanine nucleotide exchange factors (GEFs). In platelets, the GEF CalDAG-GEFI is critical for the rapid activation of Rap1b. This process is triggered by the binding of Ca^2+^ to its site on CalDAG-GEFI thus conferring GEF activity towards Rap1b [5]. Our previous work showed that phosphorylation of CalDAG-GEFI is a critical mechanism by which PKA controls Rap1b-dependent platelet aggregation [6]. CalDAG-GEFI has been extensively studied in platelets in relation to Rap1b activation, however, little attention has been paid to C3G, which also plays a role in platelet clotting through its ability to catalyse GTP-loading of Rap1b [7]. C3G is activated by interacting, via its proline-rich domain, with the SH3 domain of Crk adaptor proteins and its subsequent tyrosine phosphorylation [8, 9]. In platelets, the dominant Crk protein is Crk-like (Crkl). It is abundant and acts as an adapter for Wiskott-Aldrich syndrome protein and the tyrosine kinase Syk [8, 9]. The subsequent hydrolysis of bound GTP to GDP on Rap1b is catalyzed by GTPase-activating proteins (GAPs), and terminates the interaction between Rap1b and effector proteins controlling platelet functions. In platelets, Rap1GAP2 exhibits strong GAP activity towards Rap1b [10].

Circulating platelets are also continually exposed to inhibitory factors such as NO and prostacyclin (PGI_2_) derived from the endothelium whose effects are mediated through cGMP and cAMP, respectively [1]. It was shown that PGI_2_ and NO inhibit platelet aggregation through their ability to block Rap1b activation [11].

Whereas Rap1b controls activation of α_IIb_β_3_ integrin, VASP is involved in the inactivation of this integrin [12]. VASP belongs to the Ena/VASP protein family and is an important regulator of cytoskeletal dynamics linking cyclic nucleotide-dependent pathways to actin remodelling [13]. Evidences for a negative role of VASP in the regulation of integrins come from studies using platelets derived from VASP knock out mice. It was shown that cAMP- and cGMP-mediated inhibition of platelet aggregation is significantly reduced in VASP-null platelets [14] and VASP-null platelets exhibit augmented aggregation to endothelial cells in vivo [15]. How VASP negatively controls platelet integrins is not known.

Since activation of α_IIb_β_3_ integrin is dependent on Rap1b, we hypothesized that VASP may be a negative regulator of α_IIb_β_3_ integrin through its ability to inhibit Rap1b. To test this hypothesis, we compared activation of Rap1b in platelets from VASP-null and wild type mice in response to platelet agonists. We also investigated whether VASP interacted with Crkl in order to understand the mechanism by which VASP could regulate cyclic nucleotide-mediated regulation of Rap1b and platelet aggregation.

## Methods

### General reagents

Anti-VASP (sc-46668), anti-Crkl (sc-319), and anti-Rap1b (sc-65) Abs were obtained from Santa Cruz Biotechnology; anti-CalDAG-GEFI and anti-Rap1GAP2 Abs were from ImmunoGlobe GmbH (Himmelstadt, Germany); anti-VASP-pSer235 (clone 16C2) was from nanoTools (Teningen, Germany). Alexa Fluor 633 phalloidin (Invitrogen) was used to visualize actin filaments. The protease inhibitor cocktail was purchased from Roche.

Plasmids encoding the GST-Crkl fusion proteins were a generous gift of Dr. Stephan Feller (Martin-Luther-University Halle-Wittenberg, Germany) and proteins were purified on Glutathione-conjugated sepharose as detailed previously [16]. Recombinant His_6_-tagged human VASP was purified from *E.coli* cultures and VASP was phosphorylated by PKA in vitro as previously described [16]. Protein purities and concentrations were determined by Coomassie blue staining using bovine serum albumin (BSA) as reference.

### Rap1b pull-down assays

Platelets (2 × 10^8^) were lysed with an ice cold buffer composed of 50 mM Tris-HCl (pH 7.4), 1 % Triton X-100, 100 mM NaCl, 10 mM MgCl_2_, 20 % glycerol, 1 mM Na_3_VO_4_, and 1 mM pefabloc. The GST-RalGDS fusion protein, coupled to glutathione-sepharose beads, was used to pull down GTP-bound Rap1b as described [17]. The beads were subsequently re-suspended in 50 μl Laemmli sample buffer supplemented with 3 μl of 1 M DTT. The samples were subjected to 12 % SDS-PAGE, and transferred to PVDF membranes. The membranes were blocked in phosphate-buffered saline (PBS) supplemented with 0.2 % Tween 20 and 3 % milk, and then incubated overnight at 4 °C with an anti-Rap1 Ab (1 μg/ml dilution). After three washes with lysis buffer, the membranes were subsequently incubated for 1 h with goat peroxidase-conjugated anti-rabbit IgGs (1:10 000). The blots were again washed and antibody binding was visualised by enhanced chemiluminescence.

### Immunoprecipitations and GST pull-down assays

For immunoprecipitation, human platelets (2 × 10^8^) were lysed in the following buffer: 40 mM Hepes-NaOH, pH 7.5, 90 mM NaCl, 1 % Igepal CA-630, and a protease inhibitor cocktail. Lysates were clarified by centrifugation for 10 min at 16,000 x g at 4 °C, and Crkl was subjected to immunoprecipitation using either anti-Crkl specific Abs or isotype control Abs, followed by incubation with protein G-conjugated sepharose beads. After extensive washing with the lysis buffer, the precipitated material was analyzed by Western blotting using anti-Crkl or anti-VASP Abs.

For pull-down assays using GST-fusion proteins, human platelets (2 × 10^8^) were either treated with a combination of forskolin (FSK) (5 μM) and okadaic acid (OA) (1 μM) for 20 min at 37 °C to stimulate PKA activity (+PKA) or left untreated (−PKA). Thereafter, cells were lysed as described above. Clarified lysates were incubated with 20 μg GST-Full-length-Crkl (GST-FL-Crkl) or equimolar amounts of GST-Crkl-SH2, GST-Crkl-SH3N, GST-Crkl-SH3C, GST alone (negative control), or GST-Spec-SH3 (positive control) coupled to glutathione sepharose beads. GST pull-down experiments with recombinant VASP were performed as above, using 500 ng of in vitro PKA-phosphorylated or non-phosphorylated His_6_-VASP and 5 μg GST-FL-Crkl or equimolar amounts of the other fusion proteins in the presence of 25 ng/μl BSA to block unspecific interactions. After extensive washing of the pelleted glutathione sepharose beads, precipitated material was analyzed by Western blotting using anti-VASP Abs.

### Confocal microscopy

Washed human platelets (9 × 10^6^) were seeded on fibronectin-coated (25 μg/ml) 8-well glass bottom μ-slides (Ibidi) and fixed with 4 % formalin after 2, 5, 12, and 30 min incubation at room temperature. Cells were extensively washed with PBS, blocked/permeabilized with 5 % normal donkey serum in PBS containing 0.2 % Triton X-100, and incubated with 1:80 diluted primary Abs in 0.1 % BSA in PBS containing 0.2 % Triton X-100. After extensive washing, platelets were incubated with Alexa Fluor 488/546 conjugated donkey anti-mouse or donkey anti-rabbit Abs (Invitrogen), washed again, and mounted with 50 % glycerol in PBS containing 5 % DABCO (Sigma-Aldrich) as an anti-fading agent. Stained sections were investigated using a confocal laser scanning microscope (LSM 780, Zeiss) equipped with a 63-fold oil immersion objective. Images were acquired and prepared for presentation using the ZEN software (Carl Zeiss, version 2.1, black, 64 bit).

## Results

### Agonist-induced Rap1b activation is reduced in VASP-null platelets

**Fig. 1:**
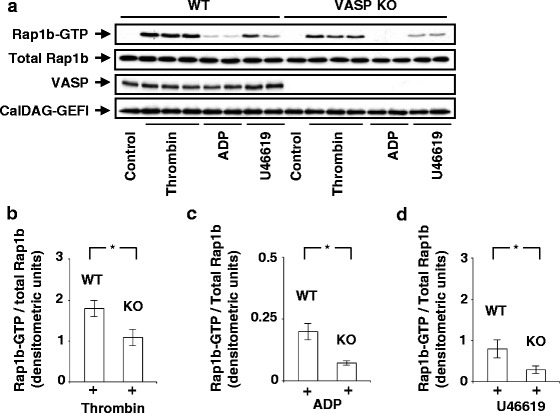
Agonist-induced Rap1b activation is reduced in VASP-null platelets. **a** Platelets (2 x10^8^) from wild type (WT) or VASP-null platelets (VASP KO) were unstimulated (control) or stimulated with thrombin (0.01 U/ml, 30s), ADP (10 μM, 1 min), or U46619 (1 μM, 1 min) (non-aggregating conditions). Thereafter, platelets were lysed and GST-RalGDS-RBD pull-down assays were performed as described in Methods. Proteins bound to GST-RalGDS-RBD were separated by 12 % SDS-PAGE, transferred to PVDF membranes, which were subjected to immunoblotting with anti-Rap1b Abs. The insets show representative Western Blots. Levels of Rap1b, VASP, or CalDAG-GEFI in the whole lysates used for the Rap1b pull-down assays were measured by Western blot analysis using appropriate Abs. The diagrams (**b**-**d**) illustrate densitometric analysis of the relative activities of Rap1b. Levels of Rap1b-GTP/total Rap1b in WT and VASP KO platelets stimulated with thrombin (**b**) (*n* = 5), ADP (**c**) (*n* = 3), or U46619 (**d**) (*n* = 3) were quantified by densitometry analysis using ImageJ software. *P < 0.05

Washed platelets from wild-type (WT) or VASP-deficient mice (VASP KO) were stimulated with thrombin, the purinergic receptor agonist ADP, or the thromboxane A2 receptor agonist U46619 and Rap1b activation was measured by GST-RalGDS-RBD binding assay. We have previously shown augmented basal Rap1a activity in neutrophils from VASP-null mice [17]. To our surprise, we observed that activation of Rap1b in response to thrombin (0.01 U/ml, 30s), the purinergic receptor agonist ADP (10 μM, 1 min), or the thromboxane A2 receptor agonist U46619 (1 μM, 1 min) was significantly decreased in VASP-null platelets in comparison to wild type platelets (Fig. 1a). The decrease of Rap1b activation in VASP-null platelets (versus wild type platelets) was 1.9-fold with thrombin (*n* = 5, *p* < 0.05), 2.8-fold with ADP (*n* = 3, *p* < 0.05) and 2.7-fold with U46619 (*n* = 3, *p* < 0.05) (Figs. 1b-d). We excluded that impaired Rap1b activation in VASP-null platelets was due to changes in the expression levels of CalDAG-GEFI, the dominant GEF expressed in platelets [5]. Indeed, genetic deletion of VASP did not affect the level of CalDAG-GEFI expression (Fig. 1a, lower blot).

### Translocation of CalDAG-GEFI and Rap1GAP2 is independent of VASP

**Fig. 2:**
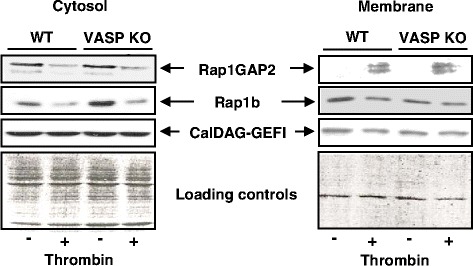
Translocation of CalDAG-GEFI and Rap1GAP2 is independent of VASP. Platelets from wild-type (WT) or VASP-deficient mice (VASP KO) stimulated or not with thrombin (0.01 U/ml, 30s) were broken by sonication in detergent-free buffer. Lysates were spun at 100,000 g for 1 h. Cytosolic (supernatant) and crude membrane fractions (pellet) were collected and re-suspended in Laemmli buffer. Proteins were separated by 12 % SDS-PAGE, transferred to PVDF membranes, which were subjected to immunoblotting with one of the following Abs: anti-Rap1GAP2, anti-Rap1b or anti-CalDAG-GEFI. The Western blots show the results of one representative experiment (out of three). Protein loading controls (Ponceau S red staining of the blots) are shown in the bottom panels

Activation of monomeric GTPases is associated with their movement, together with their regulators (GEFs and GAPs), from the cytosol to the membrane fraction. We next investigated whether VASP controlled the movement of Rap1b, CalDAG-GEFI or Rap1GAP2, the dominant GAP for Rap1b in platelets [10]. Stimulation of wild type or VASP-null platelets with thrombin (0.01 U/ml, 30s) led to a similar reduction of Rap1b in the cytosol, however, we could not detect augmented levels of Rap1b in the membrane fraction (Fig. 2). This can be explained by the fact that the majority of Rap1b (90 %) is present in the inner face of the membrane in resting platelets [18]. We observed similar movement of Rap1GAP2 from the cytosol to the membrane fraction in response to thrombin in both wild type and VASP-null platelets (Fig. 2). In contrast, we did not detect any translocation of CalDAG-GEFI from the cytosol to the membrane fraction in response to thrombin regardless of VASP expression (Fig. 2). These experiments indicate that impaired activation of Rap1b in response to thrombin in VASP-null platelets is not due to defects in the redistribution of Rap1b, Rap1GAP2, or CalDAG-GEFI between cytosolic and membrane fractions.

### NO-dependent inhibition of Rap1b is not impaired in VASP-null platelets

**Fig. 3:**
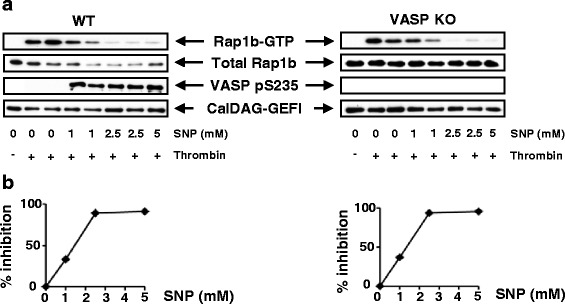
NO-dependent inhibition of Rap1b activation is not impaired in VASP-null platelets. **a** Platelets (2 × 10^8^) from WT mice (left panel) or VASP KO mice (right panel) were pretreated for 2 min with SNP (1–5 μM) and then stimulated with thrombin (0.01 U/ml, 30s). Cells were lysed and the levels of Rap1b-GTP/total Rap1b were measured as described in the legend to Fig. 1. PKG-mediated phosphorylation of VASP at serine 235 (pS235-VASP) and levels of CalDAG-GEFI in whole lysates were measured using VASP phospho-specific or anti-CalDAG-GEFI Abs, respectively. The Western blots show the results of one representative experiment (out of three). **b** Quantification of the percent inhibition by SNP (1–5 μM) of thrombin-induced activation of Rap1b in both WT and VASP KO platelets. Please note that the blot in panel A (right) was more exposed than the blot in panel A (left)

In addition to activating factors, platelets circulating in vivo are continuously exposed to inhibitory factors such as endothelium-derived NO and prostacyclin [1]. Most, if not all, inhibitory effects of NO on platelets are mediated by the soluble guanylyl cyclase/cGMP/PKG pathway [19]. Interestingly, suppression by NO of platelet tethering and adhesion to the injured vessel wall in vivo is less pronounced in the absence of VASP [15], indicating that phosphorylation of VASP by PKG may control inhibition of Rap1b. To test this hypothesis, we pre-treated mouse platelets with sodium nitroprusside (SNP), a NO-releasing drug, which strongly induces PKG-mediated VASP phosphorylation at serine 235 (Fig. 3a, third blot from the top), equivalent to serine 239 in human VASP, before stimulation with thrombin. Although the overall Rap1b-GTP levels were reduced in VASP-deficient platelets, we observed a similar SNP-mediated inhibition of thrombin-induced Rap1b activation in VASP-null and wild type platelets (Fig. 3a and b).

### Crkl and VASP dynamically interact in platelets

**Fig. 4:**
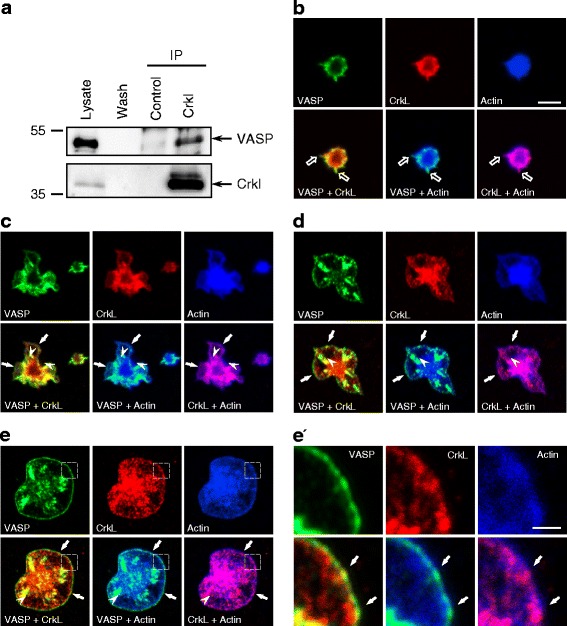
Crkl and VASP dynamically interact in human platelets. (**a**) Lysates of human platelets were immunoprecipitated (IP) with Abs against Crkl or isotype control antibodys (control) as described in Methods. Lysate, wash fraction (Wash), and precipitated material were analyzed by Western blotting with anti-VASP (upper panel) or anti-Crkl Abs (lower panel), respectively. The arrows on the right hand side indicate the position of VASP and Crkl. One representative experiment (out of three) is shown. B-E, Washed human platelets were seeded onto fibronectin-coated glass slides for 2 min (**b**), 5 min (**c**), 12 min (**d**), or 30 min (**e**) before fixation and staining with anti-VASP-specific Abs (*green*), anti-Crkl-specific Abs (*red*) or with fluorescent conjugated phalloidin to visualize actin fibers (*blue*). The top panels in each section show individual stainings; lower panels show co-localization staining of VASP and Crkl, VASP and actin, and Crkl and actin. Black arrows, white arrowheads, and white arrows indicate filopodia-, focal adhesion-, and lamellipodia-like membrane protrusions, respectively. All images have a dimension of 20 × 20 μm to better visualize platelet spreading, scale bar in B is 5 μm. In **e´**, magnified views of the indicated areas in **e** are shown; scale bar 1 μm

C3G is a GEF for Rap1b, which is activated through its recruitment by the docking protein Crkl. In adherent cells, VASP is localized to actin rich structures including the integrin-based focal adhesions and membrane protrusions such as filopodia, lamellipodia, and ruffles [13, 16, 20]. Crkl was also reported to localize to focal adhesions in mouse embryonic fibroblasts and COS7 cells [21, 22]. We asked whether VASP and Crkl could form protein complexes in platelets. To experimentally address this question, we immunoprecipitated Crkl from platelet lysates and analyzed the precipitated material by Western blot analysis using anti-VASP specific Abs. We found that the anti-Crkl Abs, but not the isotype control Abs, specifically precipitated VASP from platelet lysates, indicating an interaction between VASP and Crkl in living cells (Fig. 4a). We further confirmed such interaction between Crkl and VASP in other tissues and cells including PLB-985 cells differentiated into neutrophil-like cells and mouse spleen (data not shown).

Crkl-VASP interaction was further analysed by confocal immunofluorescence microscopy in platelets seeded on fibronectin-coated glass slides. To address the localization of Crkl and VASP during the dynamic adhesion and spreading of the platelets, cells were fixed 2, 5, 12, and 30 min after seeding and then processed for staining using anti-Crkl and anti-VASP specific Abs as well as fluorescent- conjugated phalloidin to stain actin fibres (Fig. 4b). Two min after seeding, most platelets still displayed a round morphology with some actin-rich protrusions reminiscent of filopodia extensions. Actin, VASP, and Crkl co-localized in these membrane protrusions (black arrows indicate filopodia-like protrusions) as indicated by the yellow/orange (Crkl and VASP), cyan (VASP and actin), or magenta colour (Crkl and actin) of the merged images (Fig. 4b, lower panels). As a control, we showed that no staining was observed when non-relevant primary Abs were used (data not shown). Five min after seeding, the morphology of the platelets had changed dramatically and the cells displayed a jagged shape with nascent lamellipodia-like extensions and primitive focal adhesions (arrows in Fig. 4c indicate lamellipodia-like protrusions, arrowheads indicate the proximal part of focal-adhesion-like structures). Crkl and VASP co-localized with actin at the leading edge of the lamellipodia and at the proximal end of the focal adhesions (Fig. 4c). Twelve min after seeding, the platelets were again not fully spread, however, we observed a pronounced formation of more maturated lamellipodia and focal adhesions. Again, Crkl and VASP co-localized at the leading edge of lamellipodia and at the base of focal adhesions (Fig. 4d). Thirty min after seeding, the cells were almost fully spread and a continuous lamellipodium was observed surrounding the entire cells (Fig. 4e). Magnified views of the lamellipodium revealed that VASP was mostly concentrated at the distal part of the leading edge, whereas the overlapping F-actin and Crkl showed a broader distribution and extended more proximally within the lamellipodium (Fig. 4e´). We concluded that Crkl and VASP form complexes at sites of high actin turnover, including focal adhesion-, filopodia-, and lamellipodia-like protrusions in platelets.

### The N-terminal SH3 domain of Crkl is involved in direct VASP-Crkl interaction

**Fig. 5:**
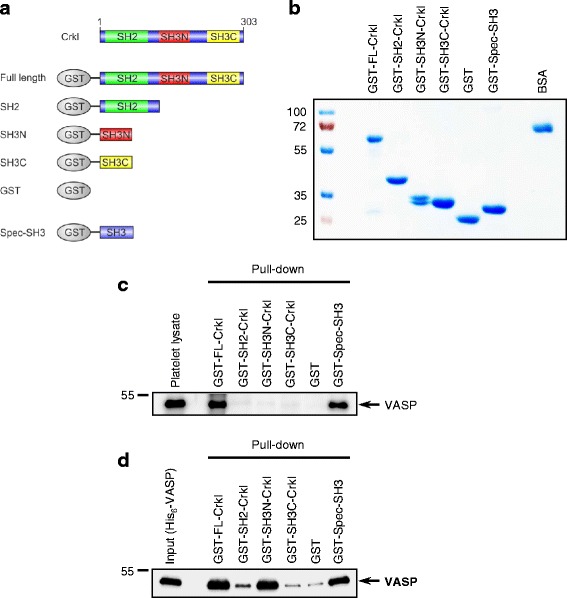
The N-terminal SH3 domain of Crkl interacts directly with VASP. **a** schematic diagramm of the Crkl domain organization and the different GST-Crkl and GST-Spec-SH3 fusion proteins used in this study. **b** Coomassie blue-stained gel of the purified GST-fusion proteins depicted in (**a**). BSA (4 μg) was loaded onto the same gel to allow relative quantification of the purified proteins. **c**, **d** GST pull-down assay with lysates of human platelets (**c**) or recombinant, purified His_6_-VASP (**d**). Equal amounts of platelet lysate (**c**) or recombinant VASP (**d**) were incubated with equimolar amounts of the depicted, immobilized GST fusion proteins or GST alone. After extensive washing, precipitated material was analyzed by Western blotting with anti-VASP specific Abs. The position of VASP is indicated on the right hand side by an arrow. The Western blots show the results of one representative experiment (out of five)

The adapter protein Crkl is composed of a N-terminal SH2 domain followed by two SH3 domains, SH3N and SH3C, respectively [23]. We have previously shown that the αII-Spectrin-SH3 domain directly binds to the proline-rich region of VASP [16, 24]. Therefore, we hypothesized that one or both of the Crkl SH3 domains may be involved in direct interaction with VASP. To test this hypothesis, we expressed and purified a series of GST-Crkl fusion proteins comprising the full length protein (GST-FL-Crkl), the SH2 domain (including the linker region between the SH2 and the SH3N domain; GST-SH2-Crkl), or each of the SH3 domains, SH3N (GST-SH3N-Crkl) or SH3C (GST-SH3C-Crkl). As negative and positive controls, we used GST alone or the SH3 domain of αII-spectrin (GST-Spec-SH3), respectively. These different constructs are described in detail in Fig. 5a and b. When we incubated a platelet lysate with equimolar amounts of the purified GST-fusion proteins described in Fig. 5a, GST-FL-Crkl and the spectrin SH3 domain bound to VASP, but not GST-SH2-Crkl, GST-SH3N-Crkl, or GST-SH3C-Crkl (Fig. 5c). This result was unexpected because SH2 and SH3 domains are known to normally fold independently of external cues. This indicates that the Crkl-VASP interaction either requires the entire 3D structure of the Crkl protein for docking or alternatively that VASP and Crkl are bridged together through a third, so far unknown protein. To experimentally address this question, we repeated the pull-down experiments with recombinant His_6_-VASP, purified from *E.coli*. We found that GST-FL-Crkl and GST-SH3N-Crkl efficiently precipitated recombinant His_6_-VASP. We also observed a weak but distinct binding of VASP to the GST-SH2-Crkl fusion protein, but interaction of VASP with GST-SH3C-Crkl was negligible. Furthermore, we found that binding of His_6_-VASP to GST-FL-Crkl, GST-SH3N-Crkl, and GST-Spec-SH3 was comparable (Fig. 5d). Together, this demonstrates that VASP interacts directly with Crkl and suggests that the SH3N domain of Crkl is involved in the binding.

### PKA-mediated VASP phosphorylation abrogates Crkl-VASP interaction

**Fig. 6:**
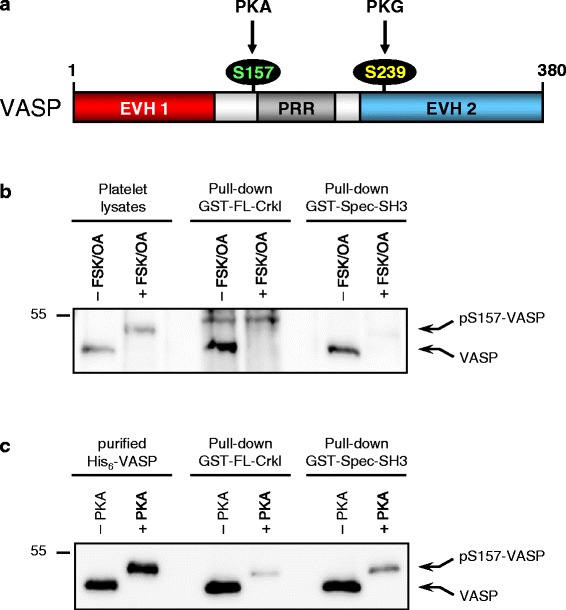
PKA-mediated VASP phosphorylation abrogates Crkl-VASP interaction. **a** Domain organization and phosphorylation sites of human VASP (380 aa); EVH1/2 Ena/VASP homology 1/2 domain; PRR, proline-rich region. VASP is a prominent substrate of cyclic nucleotide-dependent serine/threonine kinases. Human VASP is preferentially phosphorylated by PKA at serine 157 (S157, green) and by PKG at serine 239 (S239, yellow). Please note that S157 is located in close proximity to the PRR, which is important for SH3-domain mediated interactions. **b** Human platelets were stimulated or not with a combination of FSK and OA to induce PKA-mediated VASP phosphorylation at Ser157 (pS157-VASP). Equal amounts of lysates from unstimulated or stimulated platelets were incubated with immobilized GST-FL-Crkl or GST-Spec-SH3 and interaction of VASP with the GST-fusion proteins was determined as described in the legend to Fig. 5c and d. **c**. Equal amounts of purified His_6_-tagged VASP were S157-phosphorylated in vitro by PKA (+PKA) or left untreated (−PKA) and pulled-down with immobilized GST-FL-Crkl or GST-Spec-SH3. Interaction of VASP wih the GST-fusion proteins was determined as described in the legend to Fig. 5c and d. Please note that VASP Ser157-phosphorylation (but not S239-phosphorylation) induces a shift in the apparent molecular weight from 46 to 50 kDa

Signalling via PKA is important for maintaining platelets in a resting state and it was shown that PKA activity downregulates Rap1b-mediated integrin activation and platelet aggregation, at least in part, through phosphorylation of CalDAG-GEFI [6]. Since VASP is the major cytoskeletal-associated PKA substrate in platelets [25], we investigated whether PKA-mediated VASP phosphorylation regulates the interaction with Crkl. To test this hypothesis, we incubated washed human platelets with a combination of FSK and OA to stimulate PKA-dependent VASP phosphorylation at Ser157 (pS157-VASP) and used the stimulated and non-stimulated platelet lysates for pull-down assays with GST-FL-Crkl. Because PKA-mediated VASP phosphorylation at Ser157 induces an electrophoretic mobility shift of the protein from 46 to 50 kDa in SDS-PAGE, VASP specific Abs can be used to detect the phosphorylation event (Fig. 6b and c) as shown previously by us [20]. As control, we again used the SH3 domain of αII-spectrin, because we have previously shown that its interaction with VASP is sensitive to VASP Ser157 phosphorylation [16]. Strikingly, although equal amounts of stimulated and non-stimulated platelet lysates were used in the pull-down assays, only non-phosphorylated VASP protein bound to GST-FL-Crkl (Fig. 6b). We also performed pull-down assays with recombinant VASP which had been phosphorylated or not *in vitro* by PKA. Again only the non-phosphorylated protein was precipitated by GST-FL-Crkl (Fig. 6c), demonstrating that PKA-mediated VASP phosphorylation abrogates Crkl-VASP complex formation. Thus, both PKA-mediated phosphorylation of CalDAG-GEFI [6] and phosphorylation of VASP may contribute to the PKA-dependent inhibition of Rap1b in platelets.

## Discussion

An important step in platelet adhesion, spreading, and aggregation is the activation of the platelet integrin α_IIb_β_3_, which is regulated by Rap1b. In the present study, we investigated whether VASP was involved in the regulation of Rap1b in platelets. The rationale for this study was that we have previously shown augmented basal Rap1a activation in neutrophils from VASP-null mice [17] and therefore we expected a similar increase in GTP-bound Rap1b in platelets from VASP-deficient mice. To our surprise, Rap1b activation in response to thrombin, ADP, and U46619 was significantly decreased in platelets derived from VASP-null mice as compared to wild type littermates. We excluded that the observed impaired activation of Rap1b in VASP-null platelets was due to reduced expression level of CalDAG-GEFI or defects in the translocation of Rap1b, CalDAG-GEFI, and Rap1GAP2 from the cytosol to the membrane fraction during platelet activation with thrombin. We next investigated whether the NO/cGMP/PKG pathway, which negatively regulates Rap1b in platelets [11], was amplified in VASP-null platelets as this could explain decreased agonist-induced Rap1b activation in those platelets. This was not the case. Indeed, NO-dependent inhibition of Rap1b activation was similar in VASP-null and wild type platelets. This result was unexpected because VASP is abundant in platelets and is the major cytoskeletal-associated PKG substrate [25]. Thus, the antithrombotic activity of NO [26] is due, at least in part, to inhibition of Rap1b [11] but is not mediated through phosphorylation of VASP. Therefore, we propose that NO inhibits Rap1b independently of VASP via activation of GAPs, which accelerate the hydrolysis of GTP and therefore terminate the activation of Rap1b. Accordingly, NO/cGMP/PKG-dependent phosphorylation of Rap1GAP2 has been shown to increase the GTPase activity of Rap1GAP2 [10].

Since VASP is not involved in the movement of the regulators of Rap1b, and does not play a role in the NO/cGMP/PKG-dependent inhibitory pathway, we next investigated whether VASP was involved in an alternative pathway of Rap1b regulation, in which a GEF different from CalDAG-GEFI could be involved. In this regard, Gutiérrez-Herrero and co-workers [7] have demonstrated, by using transgenic mouse models that overexpress C3G or C3GΔCat (a dominant negative mutant that lacks catalytic activity), that C3G increases platelet activation and aggregation both in vitro and in vivo through its ability to catalyse GTP-loading of Rap1b. C3G is a GEF for Rap1b which is activated through recruitment by the docking protein Crkl [8]. Crkl, which is abundant in platelets [9], contains two SH3 domains (SH3N and SH3C) and one SH2 domain [8]. We demonstrated that VASP is dynamically interacting with Crkl. Our binding studies with platelet lysates and recombinant VASP protein demonstrated that the VASP-Crkl interaction is direct and involves the Crkl SH3N domain. By interacting with Crkl, VASP may transfer this protein to the platelet cytoskeleton by means of interaction with F- or G- actin [13] and this would explain, at least in part, association of Crkl with the cytoskeleton in platelets [9]. Accordingly, we did not find Crkl in the membrane fraction of wild type or VASP-null platelets (data not shown) and we showed that Crkl and VASP form complexes at sites of high actin turnover, including focal adhesion-, filopodia-, and lamellipodia-like protrusions during platelet spreading. Crkl has been shown to recruit tyrosine kinases, such as Syk, present in the cytoskeleton of activated platelets [9]. Such recruitment of Crkl to actin-rich structures may be important for phosphorylation of C3G bound to Crkl and the subsequent activation of Rap1b.

We made novel findings regarding the domains of VASP and Crkl involved in complex formation. We found that recombinant His6-VASP, but not VASP present in platelet lysates, bound to both the GST-SH3N-Crkl domain and the GST-SH2-Crkl fusion protein (however weakly). The SH2 domain of Crkl binds to phospho-tyrosine motifs [27], but we could not detect tyrosine phosphorylation of VASP purified from E.coli (data not shown). However, when we closely examined the sequence of GST-SH2-Crkl, which also covers the linker region between the SH2 domain and the SH3N domain, we spotted a Y^105^PSPP motif (numbering in human Crkl) in the linker, which conforms to the consensus binding motif of the VASP EVH1 domain (F/L/W/Y)PxϕP (where ϕ is a hydrophobic residue) [28]. Therefore, we propose that binding of the VASP EVH1 domain to the Crkl linker may support the interaction of SH3N-Crkl with VASP. The lack of binding of VASP in platelet lysates to SH3N-Crkl may be explained by the fact that some proteins in platelet lysates compete with VASP for binding SH3N-Crkl and have a better affinity than VASP for this motif. Alternatively, because VASP forms oligomers with itself, Mena or Evl [29, 30], VASP oligomers interaction with Crkl may require not only the SH3N domain of Crkl, but also other domains including the SH3C domain and the linker region between the SH2 domain and the SH3N domain. Future studies will be aimed at testing this hypothesis.

SH3 domains are the most abundant protein recognition motifs, comprising an estimated 409 copies in the human proteosome alone [31], mostly found in signal transduction and cytoskeletal proteins. SH3 domain-mediated interactions are commonly found in processes that require the rapid subcellular recruitment or interchange of proteins during initiation of signalling cascades and cytoskeletal rearrangements [32]. Due to the insufficient inherent specificity in most SH3-mediated interactions, additional mechanisms exist in vivo to generate protein binding selectivity. These are temporal and cell-type specific gene expression, combination of multiple separate interactions between two binding partners, and the cooperative assembly of multiprotein complexes. Most common, however, is the compartmentalization of binding partners and the regulation of their interaction by posttranslational modifications such as phosphorylation [32]. In the present study, we showed that the interaction between VASP and Crkl takes place in actin-rich structures and is regulated by means of phosphorylation of VASP on serine 157. Thus, PKA-mediated phosphorylation of VASP abrogates the interaction with Crkl which may, at least in part, provide a rationale for the PKA-dependent inhibition of Rap1b activation and platelet aggregation. Serine157 is the preferred PKA phosphorylation site in VASP. Notably, this phosphorylation site is located in close proximity to the proline-rich region of VASP, which interacts with SH3 domain containing proteins. We and others have previously shown that PKA-mediated phosphorylation of VASP S157 abrogates binding of some SH3-domains of proteins, whereas binding of other SH3 domains of other proteins seemed to be independent of the phosphorylation status [16]. In contrast to the PKA phosphorylation site (S157), the preferred PKG phosphorylation site (S239) is not located in proximity to the proline-rich region of VASP, but in the EVH2 domain, adjacent to the G-actin binding motif. We have previously shown that phosphorylation at this site affects actin dynamics rather than (SH3-domain mediated) protein-protein interactions and subcellular protein targeting [20].

Platelets express five types of integrins: three β1 integrins and the two β3 integrins α_v_β_3_ and α_IIb_β_3_ [33]. The β1 integrins mediate platelet adhesion to the matrix proteins collagen, fibronectin, and laminin, whereas α_v_β_3_ and α_IIb_β_3_ bind vitronectin and the RGD motif of Von Willebrand factor, respectively [33]. In fibroblasts, engagement of α_v_β_3_ leads to PKA-dependent phosphorylation of VASP which drives suppression of β1 integrin activation. This is achieved through disruption of the interaction between phosphorylated VASP and the Rap1-GTP-interacting adaptor molecule (RIAM) [34]. Based on this finding, it is plausible that absence of VASP in platelets may relieve the inhibition exerted by α_v_β_3_ on β1 integrins, thus explaining the augmented adhesive capacities of VASP-null platelets to injured blood vessels [15] since we excluded any augmented agonist-induced activation of Rap1b. This implies that Rap1b not only controls integrin inside-out signalling but plays a complex role in the regulation of platelet functions as shown by other investigators reporting Rap1b control of α_IIb_β_3_ outside-in signalling as well as secretion of platelet granules [35].

In summary, we propose that the formation of a ternary C3G/Crkl/VASP complex regulates, in parallel with CalDAG-GEFI, Rap1b-dependent platelet activation and this may explain why agonist-induced activation of Rap1b in VASP-null platelets is impaired. PKA-dependent phosphorylation of VASP on serine residue 157 abrogated Crkl binding, which may provide, at least in part, a rationale for the PKA-dependent inhibition of Rap1b activation and platelet aggregation.

## Conclusions

Crkl and VASP are dynamically interacting in actin-rich domains during platelet spreading and the interaction involves the N-terminal SH3 domain of Crkl. We propose that Crkl-VASP complex formation is important for C3G-dependent Rap1b activation in platelets. Conversely, Rap1b activation in VASP-null platelets is reduced. The interaction between Crkl and VASP is regulated by PKA-mediated VASP phosphorylation. This may, at least in part, explain the PKA-dependent inhibition of Rap1b and platelet aggregation.

## Abbreviations

Abs: Antibodies
ADP: Adenosine diphosphate
Crkl: Crk-like protein
FSK: Forskolin
GAP: GTPase activating protein
GEF: Guanine nucleotide exchange factor
NO: Nitric oxide
OA: Okadaic acid
PBS: Phosphate-buffered saline
VASP: Vasodilator-stimulated phosphoprotein
